# Closing the Mental Health Access Gap Through Novel Analytics

**DOI:** 10.7759/cureus.42093

**Published:** 2023-07-18

**Authors:** Christine M Skovira, Elizabeth Pfoh, Amy Thompson, Julie Rish

**Affiliations:** 1 Department of Medicine, Michigan State University College of Human Medicine, Grand Rapids, USA; 2 Department of Clinical Transformation, Cleveland Clinic Foundation, Cleveland, USA; 3 Center for Value-Based Care Research, Cleveland Clinic Foundation, Cleveland, USA

**Keywords:** predictive modeling, access to healthcare and health outcomes of vulnerable populations, importance of treating depression, generalised anxiety disorder, psychiatry and mental health

## Abstract

Depression and anxiety are associated with substantial morbidity, including physical deterioration. Connecting individuals to timely care improves outcomes. Unfortunately, significant gaps remain between the demand for behavioral healthcare and the supply of care. Further, estimates of demand are based on retrospective and/or non-localized measures, which impedes planning. This poses an opportunity to rethink how to close this gap. Health systems are better positioned than ever to do so, given novel technologies, data, and community integration. By developing more localized, real-time models of depression and anxiety demand and healthcare supply, health systems can better prioritize resource deployment and partnerships to proactively meet patient needs.

## Editorial

Lori works in healthcare. She loves caring for patients but struggles to care for herself. Her undiagnosed depression was manageable until her partner lost their job. Her default coping mechanism, eating and drinking, spiraled as she tried to cope with the stress of lost income. As her mood dipped, her blood sugar skyrocketed. Her exhaustion made prioritizing her own health untenable.

When she did have an annual exam, her physician recognized her depression and offered her a number to call. Unfortunately, the next appointment was months away so she never followed through. After an emergency department visit for diabetes, she began dialysis. Only then did she realize the toll that her depression had taken on her physical health.

Lori is not alone. Individuals with anxiety and depression are frequently undiagnosed or under-treated, with the risk increasing in marginalized populations [1-7]. Nearly 50% of emergency department visits related to behavioral health concerns have no prior ambulatory behavioral healthcare [8]. Further, only 10-42% of diagnosed patients receive adequate treatment for anxiety and depression [9]. Average wait times for behavioral health visits are approximately 25 days for a first visit, with only 7-15% of psychiatrists accepting new patients in the next 2-4 weeks [10].

The sequelae of under-treatment for depression and anxiety are severe and growing in the United States [11-14]. In the past two decades, “deaths of despair” (e.g., mortality attributed to drug overdoses, suicide, and alcoholic liver disease) have increased between 56% and 387% [15]. Importantly, these sequelae are avoidable with appropriate treatment. Studies show that timely follow-up care for behavioral health concerns is associated with a significantly lower risk of suicide [16].

There are key barriers to timely anxiety and depression treatment. For patients, lack of access (e.g., wait times, geographic distance, insurance coverage, out-of-pocket cost) is dominant [17]. For example, patients are more likely to postpone or forego treatment if timely access to a behavioral health specialist is unavailable [18-19]. For health systems, providing access is an ongoing challenge. Behavioral health professional shortages are seen in every state, with the largest gaps in rural and low-income areas. Furthermore, psychiatry is one of the oldest specialty workforces [20]. As such, health systems must efficiently marshal the limited resources they have to close access gaps. However, methods for doing so remain unclear.

Given the need, for-profit companies have launched with the goal of improving access. Behavioral health-focused digital health technologies raised more venture capital funds than any other clinical indication in 2021 at $3.3B, double the investment seen in 2020 [21]. While overall healthcare venture capital decreased in 2022, behavioral health applications were again the highest-funded focus area in digital health at $1.3B [22]. The focus of behavioral healthcare technology companies is increasingly diverse. Companies such as BetterHelp, TalkSpace, and Teladoc focus on connecting subscribers to on-demand, text-based therapy. BetterHelp, for example, claims to facilitate over 5 million behavioral health video sessions, voice calls, chats, and messages every month [23]. Other companies focus on providing access for specific populations, including individuals with obsessive-compulsive disorder (e.g., NOCD), individuals with eating disorders (e.g., Equip Health, Arise), individuals with substance use disorders (e.g., Workit Health, Bicycle Health), students (e.g., Mantra Health), and youth (e.g., Little Otter, Brightline). Many of these companies offer free screening and same-day appointments virtually. In doing so, these offerings seek to close gaps and prevent deferrals of care.

These companies provide novel benefits such as convenient, same-day therapeutic options within patients’ home states. However, these treatment touch-points are not typically associated with the health systems where patients receive their healthcare. This fragmentation may result in poorer quality of care due to suboptimal care continuity and coordination. Further, disadvantaged populations may be systematically less likely to access these services given widespread digital poverty [24] and the need to pay out-of-pocket for many of these services. Thus, individuals at the highest risk of depression and anxiety may be less likely to access these technologies and to access mental healthcare more broadly [25-31].

Given their clinical expertise, community knowledge, and data, health systems are uniquely positioned to link patients with evolving behavioral health resources for anxiety and depression. Yet, health systems must first equip themselves with further enhanced decision-making tools. In particular, healthcare systems have all the inputs necessary to build powerful predictive models of behavioral health needs. Predictive modeling uses data processing and analytics to identify patterns in data as well as the explanatory variables that drive patterns. While predictive modeling has advanced significantly in community screening for other conditions, few tools exist for quantifying near-term expected changes in the community-level prevalence of anxiety and depression. Current demand and supply approaches inform health systems’ ideal next steps for such tool development.

Demand

In the current state, demand involves health system-centric and/or non-localized estimates. Health systems typically model demand based on the number of patients who accessed the health system in a prior period. This approach, based on electronic health record data, vastly underestimates the need since not all individuals can or do access healthcare. While population-level surveys, such as the Census Bureau’s American Community Survey, may provide more population-driven input, such data is retrospective and not system-specific, thus limiting its utility for more real-time adjustments to system-level capacity planning. The Health Resources and Services Administration (HRSA) and Substance Abuse and Mental Health Services Administration (SAMHSA)’s Health Workforce Simulation Model (HWSM) provides an initial approach for projecting future needs based on diverse population-level inputs. These inputs can include demographic, socioeconomic, health status, health risk, and insurance information using data from the Census Bureau American Community Survey, the National Nursing Home Survey, the Behavioral Risk Factor Surveillance System, and the Urban Institute’s state-level estimates of the impact of the Affordable Care Act [32]. Despite using these novel inputs, output models like the HWSM are still often retrospective and presented at aggregate levels. This presents challenges in using such models for localized health system capacity planning.

In an ideal state, demand should be estimated using syndemic, patient-centric models. Health systems should seek to anticipate patient needs in more real-time using underlying drivers of anxiety and depression such as economic position or living situation. This approach enables health systems and partners to foresee when key drivers (e.g., unemployment) or proxy behaviors (e.g., alcohol consumption) are rising in a local community and to prepare for the increasing prevalence of anxiety and depression. To do this, health systems must engage with traditional partners, such as local community centers, as well as less common partners, such as community health workers and novel technology companies, to understand changing community dynamics. By understanding expected changes in anxiety and depression, capacity planners can better prioritize mitigation strategies to connect patients to care more efficiently and remain flexible when the community influences change.

Supply

Currently, estimations of provider supply are largely based on provider graduation rates. Supply modeling introduces unique challenges in behavioral health, given fragmented access points. Patients may receive treatment from various community, governmental, or healthcare sources such as church, school, or a local independent healthcare provider. Patients may even receive behavioral health support from multiple providers across primary and specialty care within a system. Fortunately, the HRSA's HWSM has shown how supply can be modeled, including a broader list of provider types such as clinical and school psychologists, substance abuse and behavioral disorder counselors, behavioral health social workers, and marriage therapists. However, these estimations are also driven by provider graduation rates, which may under- or over-estimate supply based on provider migration into or out of their graduation region. Additionally, the results of such models are available only at a national and state level.

In an ideal state, coalition-built views of real-time regional supply will be designed for operational use. Health systems can build from the HWSM’s supply modeling methodology to understand local resources. To refine estimates, health systems should collaborate regionally to create a database of existing resources that is continually updated based on localized provider attrition and migration data. Key collaborators will include other providers, government bodies, payers, community organizations, and technological innovators.

Once the discrepancy between supply and demand is known, health systems can identify and address gaps. This will enable more proactive community-oriented outreach to populations at rising risk of mental illness and enhanced capacity planning. For example, shared medical appointments, asynchronous communication tools, and expanded roles for community behavioral health workers and advanced practice providers can be allocated for communities that need increased access. Additionally, based on the tool’s findings, health systems can better prioritize and further partnerships to expand access. In the future, this approach could be expanded to other behavioral health concerns such as bipolar disorder, psychosis, and substance use disorder.

Imagine how Lori’s outcome might be different. In this scenario, her health system was aware of the economic instability in her community and converted individual appointments to shared medical appointments allowing more patients to receive care. With enhanced access, Lori was able to see someone that week. Rather than continual withdrawal and worsening health, Lori learned coping strategies to prioritize her diabetic care. Early identification and treatment preserved Lori’s health and quality of life.
